# Family communication patterns and perfectionism as predictors of academic procrastination in nursing students

**DOI:** 10.1186/s12909-026-08780-0

**Published:** 2026-02-13

**Authors:** Bahman Dashtbozorgi, Saeed Ghanbari, Esmaeil Mousavi Asl, Alireza Baghrobehbahani

**Affiliations:** 1 Diabetes Research Center, Health Research Institute, Ahvaz jundishapur university of medical sciences, Ahvaz, Iran; 2 Department of Biostatistics and Epidemiology, School of Health, Ahvaz Jundishapur University of Medical Sciences, Ahvaz, Iran; 3 Department of Psychiatry, Golestan Hospital, , School of Medicine, Ahvaz Jundishapur University of Medical Sciences, Ahvaz, Iran; 4 Student Research Committee, Ahvaz Jundishapur University of Medical Sciences, Ahvaz, Iran

**Keywords:** Academic procrastination, Perfectionism, Family communication patterns, Nursing students

## Abstract

**Introduction:**

Academic procrastination is a common challenge faced by students, and it can negatively impact their academic progress. Notably, perfectionism and family communication patterns influence and shape students’ academic behaviors. Therefore, the present study aimed to investigate and predict academic procrastination based on perfectionism and family communication patterns among nursing students.

**Methods:**

This cross-sectional descriptive-analytic study was conducted among 151 nursing students at Ahvaz Jundishapur University of Medical Sciences, selected using a stratified random sampling method. Inclusion criteria were willingness to participate and enrollment in semesters 2–7. Participants who did not complete the questionnaires were excluded. The data collection tools used in the study included the Tuckman Procrastination Questionnaire (TPS), the Ahvaz Perfectionism Scale (APS), and the Koerner and Fitzpatrick Family Communication Patterns Questionnaire (RFCP). Data were analyzed using independent t-tests, analysis of variance (ANOVA), Pearson correlation, and regression analysis. The significance level was considered to be 0.05.

**Results:**

The participants had an average age of 21.58 years, and their average GPA was 16.35. The results revealed that 64.9% of the students exhibited moderate to severe academic procrastination, while 89.4% showed moderate to severe perfectionism. Additionally, the conversation orientation mean (62.61) was higher than the conformity orientation score (41.45). Correlation analysis indicated a positive and significant relationship between academic procrastination and perfectionism (*r* = 0.416, *p* < 0.01). Furthermore, the results of the T-tests showed a significant relationship between family communication patterns and both academic procrastination (sig = 0.004, *p* < 0.01) and perfectionism (sig = 0.000, *p* < 0.01) among the students.

**Conclusion:**

Students’ academic procrastination is affected by both personal traits and the family environment. Perfectionism contributes to procrastination by creating pressure and instilling a fear of making mistakes. On the other hand, a positive family conversation orientation against conformity orientation can have a protective effect by enhancing feelings of responsibility and self-efficacy.

**Graphical abstract:**

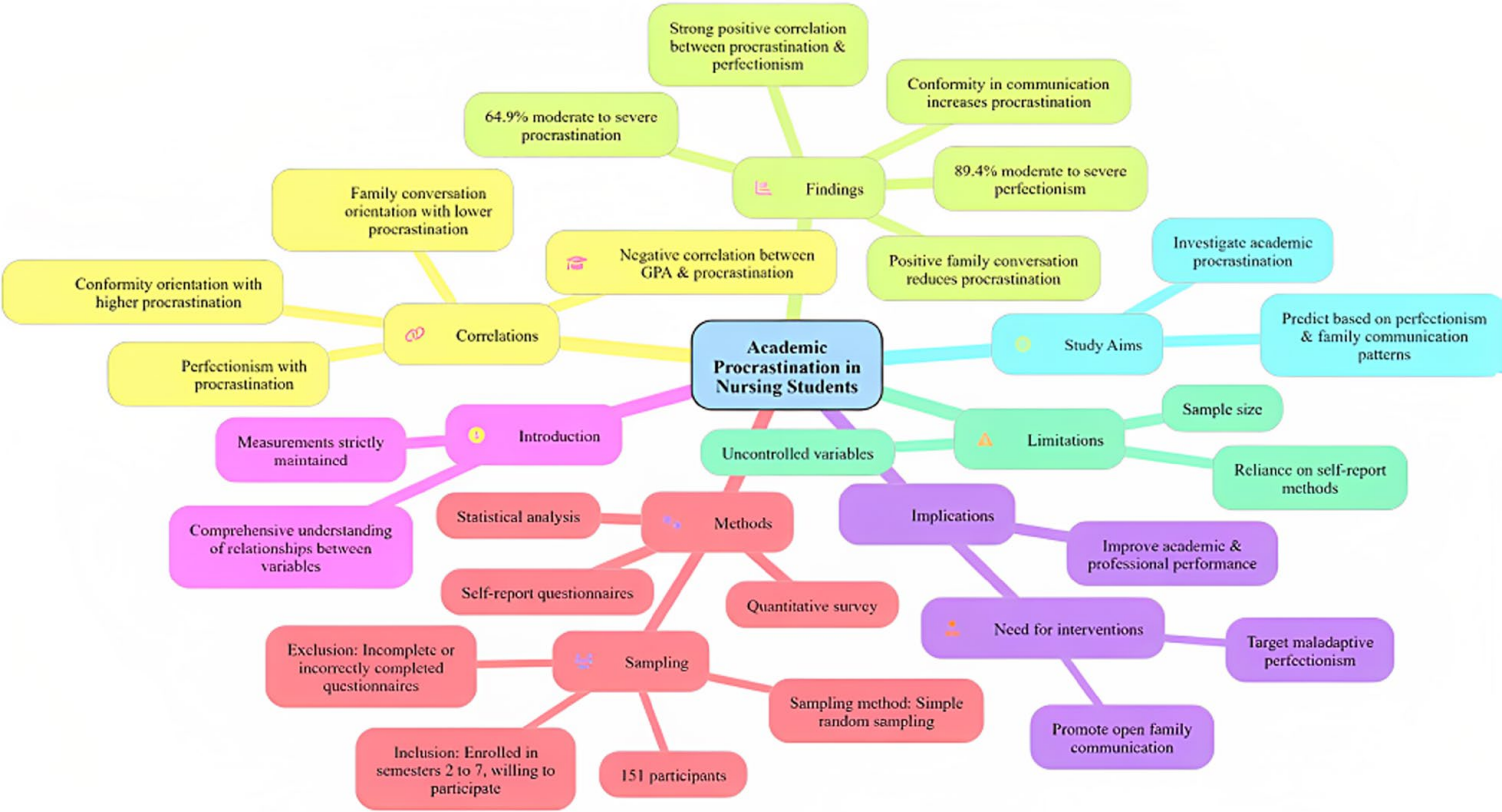

## Background

Students are the fundamental pillar of the country’s educational system, playing a crucial role in achieving educational goals. Therefore, focusing on their education enhances the productivity of the educational system [1]. In this context, academic procrastination (AP) is a common challenge that can impact students’ academic performance and lead to serious consequences for both students and the healthcare system [2]. Academic procrastination refers to the failure to complete academic tasks and assignments on time [3], which is the focus of the present study. Regarding homework behaviors, two types of patterns have been observed among students. The first type is thoughtful and purposeful: when faced with multiple assignments, students prioritize the most important tasks. The second type is irrational and harmful, that known as academic procrastination [2, 3]. Chehrzad et al. found that 69.5% of students at that university were procrastinating [4]. Similarly, a study in China found that 65–85% of nursing students exhibited procrastination, with rates increasing each successive year of education [5]. Academic procrastination among students sends undesirable signals, leading to academic failure and probation. It also wastes students’ material and spiritual resources, as well as those of society [6]. Nursing students encounter numerous stressors, including a heavy load of clinical courses, various tests to assess their clinical and theoretical competence, social pressures, and expectations from society and their university [7, 8]. Specifically, procrastination among nursing students can lead to a reduced level of awareness and knowledge, which negatively impacts their ability to acquire essential patient care skills. Therefore, once they enter the workforce, these procrastinating students often experience heightened stress, anxiety, job burnout, medication errors, and a desire to retire early. They may also show reduced motivation for patient care and a decline in the quality of care compared to their peers [8, 9]. Overall, psychologists view academic procrastination as a multifaceted phenomenon encompassing emotional, sensory, cognitive, and behavioral aspects, with various factors, including perfectionism, influencing this behavior [10].

Perfectionism is defined as the pursuit of perfection and the establishment of extremely high performance standards, coupled with an inclination to be overly critical of oneself [11]. It represents an irrational belief that both individuals and their environments must be flawless, leading to the expectation that any undertaking should be completely free of mistakes and errors [12]. Perfectionists often assess their self-worth based on their performance and achievements. Because perfectionists set such high standards, their mindset can lead to academic procrastination [13]. Students who exhibit high levels of perfectionism experience increased anxiety and distress and tend to have lower self-esteem. These combined factors contribute to academic burnout, procrastination, and a lack of academic vitality [14]. In contrast, students with moderate to low levels of perfectionism generally report experiencing less anxiety. They tend to set achievable goals for their educational pursuits and hold reasonable expectations for their performance and actions [14, 15]. This stands in stark contrast to students with high levels of academic perfectionism, whose mindset negatively impacts important academic aspects such as procrastination and burnout [14]. Research supports the hypothesis that perfectionism develops in families where parents are highly critical of their children’s performance [16]. Frost et al. found that individuals with high levels of perfectionism often perceive their parents as demanding, authoritarian, and critical [17]. Similarly, a study by Kawamura et al. revealed that children frequently view their parents as emphasizing an authoritarian parenting style in Asian communities [18]. This suggests that perfectionism is more likely to develop in Asian families, where parents are particularly demanding and critical. However, not all evidence supports the idea that perfectionism is transmitted from one generation to the next. Some studies have indicated that perfectionistic children do not always come from perfectionistic families. Despite the significant interest in understanding perfectionism, few studies have focused on how this trait develops in individuals [19, 20]. To gain a deeper understanding of the role of the family in shaping perfectionism and academic procrastination, researchers have examined a family variable known as family communication patterns.

Family communication patterns (FCP) refer to how family members interact to develop shared beliefs and make decisions together [20]. These patterns consist of two main dimensions: conversational and conformity orientations [21]. Conversational orientation is defined as the extent to which families create an environment where all members are encouraged to freely and comfortably participate in discussions, exchange opinions on a variety of topics, and engage in open communication without time constraints [22]. In contrast, conformity orientation refers to the degree to which family members emphasize similarities in attitudes, values, conflict avoidance, and interdependence [22]. Family communication patterns are shaped by the experiences that arise from family interactions. These dimensions influence how much family members express and share their thoughts and feelings. Socio-emotional development begins with the parent-child relationship, and strong family communication support is considered essential for a child’s sense of security and self-esteem. Individuals undergo various cognitive and behavioral changes throughout the educational period, which can increase the likelihood of academic procrastination [23]. Today, nursing students are vital contributors to the country’s workforce and play a key role in the progress and development of the healthcare system. Despite extensive research on academic procrastination, few studies have simultaneously examined the role of individual traits, such as perfectionism, together with family communication patterns, particularly among nursing students. This study addresses this gap by investigating the roles of perfectionism and family communication patterns in predicting academic procrastination, aiming to provide a more comprehensive understanding of this phenomenon in nursing students.

## Materials and methods

### Study design and setting

This descriptive-analytical correlational study was conducted at Ahvaz Jundishapur University of Medical Sciences, located in southwestern Iran. This study investigated the mediating role and association of perfectionism and family communication patterns with academic procrastination in nursing students. The report of this study follows the guidelines for Strengthening the Reporting of Observational Studies in Epidemiology (STROBE) [24].

### Sampling and participants

In this study, the research population included all nursing students studying at Jundishapur University of Medical Sciences, Ahvaz, Iran. 850 nursing students were studying at Jundishapur University at the time of the study. The sample size was calculated as 151 people using the following formula:

$$n=\frac{\left(Z_{1-\alpha/2}+Z_\beta\right)^2}{\left(0.5\;ln{\displaystyle\frac{1+r}{1-r}}\right)^2}+3$$

Sampling was conducted in two stages. In the first stage, stratified sampling was used to determine the number of students to be selected from each center: Ahvaz, Ramhormoz, Masjed Soleiman, Bandar Mahshahr, and Bostan. The sampling fraction was calculated by dividing the study sample size (*N* = 151) by the total population of nursing students at the time of the study (*N* = 850), resulting in a fraction of 0.177. This fraction was then multiplied by the number of students at each center to determine the number of students to be sampled: Bandar Mahshahr (*n* = 9), Masjed Soleiman (*n* = 12), Izeh (*n* = 15), Ahvaz (*n* = 89), Ramhormoz (*n* = 13), and Bostan (*n* = 13).

In the second stage, simple random sampling was applied. First, a complete list of students at each center was compiled. Then, a random number table and an online random number generator were used to ensure that each student had an equal chance of selection. It should be noted that all eligible students were considered for random selection, and the sampling was not stratified by class or semester. Inclusion criteria required students to be willing to participate and enrolled in semesters 2 to 7. Students who did not fully complete the questionnaires were excluded. Additionally, to ensure data quality, questionnaires suspected of being completed incorrectly or negligently were either excluded or reviewed with the participant, who was asked to complete them again.

### Data collection procedure

Data collection for this study began in December 2024 and was completed in March 2025. Researchers were present at the research site to collect data, and after selecting the samples, study questionnaires were distributed to them. The participants were fully informed about the study objectives and the procedures for completing the questionnaires. Informed consent was obtained from all participants. They were given sufficient time to complete the questionnaires and were encouraged to ask any questions regarding the study process. All ethical considerations were observed throughout the study, and participants’ privacy was strictly maintained.

### Measurements

#### Tuckman procrastination questionnaire (TPQ)

The Tuckman Procrastination Questionnaire (TPQ) was utilized to assess students’ levels of academic procrastination. Developed by W. Bruce. Tuckman (1991) developed a 16-item instrument to evaluate individuals’ tendencies to delay academic tasks. Each item is rated on a four-point Likert scale ranging from 1 to 4, with 12 items scored directly and four items (questions 7, 12, 14, and 16) reverse-scored. The total score ranges from 16 to 64, with higher scores indicating greater procrastination; specifically, scores between 16 and 32 reflect low procrastination, scores between 33 and 48 indicate moderate procrastination, and scores of 49 or higher signify high procrastination [25]. Tuckman (1991) reported strong psychometric properties for this instrument, with a Cronbach’s alpha of 0.86 and a content validity index of 0.83. Subsequent studies have confirmed its reliability and validity, including research by Kazemi et al. (2010), who reported a Cronbach’s alpha of 0.71 and a validity coefficient of 0.78, and Farajzadeh et al., who reported a Cronbach’s alpha of 0.79 [26, 27]. The internal consistency of TPQ was assessed in this study using Cronbach’s alpha and showed a value of 0.911, indicating excellent reliability.

### Demographic information form

The demographic information form in this study included information on age, gender, academic semester, marital status, semester GPA, family income level, and parents’ education level.

#### The Ahvaz perfectionism scale (APS)

Najarian developed this self-report instrument, which consists of 27 items scored on a four-point Likert scale ranging from 0 to 3. All items are directly scored, except for items 11, 16, 17, and 22, which are reverse-scored. The total score ranges from 0 to 81, with scores of 0–27 indicating low perfectionism, 28–54 indicating moderate perfectionism, and above 54 indicating high perfectionism. The developers of the scale reported strong internal consistency, with a Cronbach’s alpha of 0.90 and a test–retest reliability coefficient of 0.68 for the total scale. Hormozinejad (2001) also assessed the reliability of the scale using the split-half method, reporting coefficients of 0.88 and 0.83, respectively. Furthermore, Najarian et al. established the scale’s convergent validity, reporting correlations of 0.65 with the Type A Behavioral Pattern Scale, 0.41 with the Physical Complaints Subscale, and 0.39 with the Cooper-Smith Self-Esteem Inventory, respectively, supporting its construct validity. Hormozinejad et al. also evaluated and confirmed the adequacy of the scale’s validity [28]. The internal consistency of APS was assessed in this study using Cronbach’s alpha and showed a value of 0.90, indicating excellent reliability.

#### Revised family communication patterns (RFCP)

The Koerner and Fitzpatrick Family Communication Patterns Questionnaire was used to assess family communication styles. This instrument consists of 26 items rated on a five-point Likert scale and measures two dimensions: conversation orientation (items 1–15) and conformity orientation (items 16–26). The score range for conversation orientation is 15–75, whereas that for conformity orientation is 11–55. Higher scores on each scale indicate a stronger perceived presence of that orientation within the family [29]. The questionnaire was originally developed by Fitzpatrick and Koerner (2004) and later translated into Persian by Latifian et al. (2007). Fitzpatrick and Koerner confirmed its content and construct validity and reported a Cronbach’s alpha value of 0.82. Latifian and colleagues reported a validity coefficient of 0.83 through factor analysis. Cronbach’s alpha coefficients for this questionnaire were 0.78 for conversation orientation and 0.77 for conformity orientation, indicating acceptable reliability [29, 30]. The internal consistency of the RFCP was assessed using Cronbach’s alpha, yielding a value of 0.726. The conversation and conformity orientations were also evaluated separately, resulting in Cronbach’s alpha values of 0.920 and 0.897, respectively.

### Statistical analysis

Descriptive statistics, independent t-tests, correlation analysis, and regression analysis were conducted in this study. All data analysis and the generation of graphs and tables were performed using SPSS software (version 27), with a significance level of 0.05. In this study, the normality of the dependent variable, Academic Procrastination, was assessed and confirmed using the Shapiro-Wilk test (*p* = 0.338). The normality of the regression residuals was assessed using histograms and normal probability (P–P) plots. Visual inspection revealed an approximately normal distribution, characterized by a bell-shaped histogram and P–P plot points that were closely aligned with the diagonal line. Additionally, the Durbin-Watson test (DW = 1.92) confirmed the independence of residuals, indicating that there was no significant autocorrelation.

When examining family communication patterns, it is important to note that the unequal number of items in the two subscales, conversation orientation and conformity orientation, makes it impossible to directly compare their raw scores. To solve this problem and minimize the impact of the different number of options in measuring communication patterns according to the questionnaire, the following formula was used to equalize the weight of the scores [31]:

$$\begin{aligned}&Standardized\;Score\;\\&=\left(\frac{\left(Raw\;Score-Minimum\;Score\right)}{\left(Maximum\;score-Minimum\;Score\right)}\right)\;\ast100\end{aligned}$$

To categorize the levels of perfectionism and procrastination in this study, the guidelines and scoring methods of the respective questionnaires were followed. Both variables were divided into three levels: low, medium, and high. For family communication patterns, a score was assigned to each dimension, with higher scores indicating greater prominence of that communication pattern within the participant’s family. Therefore, the scores were standardized using the above formula to account for differences in the number of items, enabling more accurate comparison and classification, and providing a clearer understanding of the subject.

## Results

### Demographic findings

57.6% of participants (*n* = 87) were male, and 42.4% (*n* = 64) were female. The mean age of the nursing students was 21.58 ± 1.95 years, and their mean cumulative grade point average (GPA) was 16.35 ± 1.47. Most students (97.4%) were single. Regarding family income, the majority of participants (47%) reported having an average financial status.

### Descriptive findings about study variables

Means and standard deviations of the score of academic procrastination among nursing students were (36.00 ± 9.02), which was at a moderate level. According to the scoring system of the Tuckman Procrastination Questionnaire (TPQ), 58.3% (*n* = 88) of students demonstrated moderate procrastination, while 6.6% (*n* = 10) exhibited severe procrastination. Means and standard deviations of the score of perfectionism, among nursing students, were (42.97 ± 12.25), which indicated a moderate level among students. According to the scoring system of the Ahvaz Perfectionism Scale (APS), 70.9% (*n* = 107) reported moderate perfectionism, and 18.5% (*n* = 28) reported severe perfectionism. Regarding family communication patterns, according to the Revised Family Communication Patterns (RFCP) questionnaire, the results showed that the mean and standard deviation of the score for the conversation orientation were (62.61 ± 19.41), and the conformity orientation was (41.45 ± 21.21). Considering the equalization of the weight of the scores obtained in communication patterns, 70.9% of students reported conversational orientation as the dominant communication pattern in their families (Table 1).

**Table 1:** Means and standard deviations of the scores of academic Procrastination, Perfectionism, conversation orientation, and conformity orientation

Variable	Mean ± SD	Percentage
Academic procrastination	36.00 ± 9.02	Low = 35.1%
		Moderate = 58.3%
		severe = 6.6%
Perfectionism	42.97 ± 12.25	Low = 10.6%
		Moderate = 70.9%
		Severe = 18.5%
Conversation orientation	62.61 ± 19.41	70.9%
Conformity orientation	41.45 ± 21.21	29.1%

### Analytical findings

Table 2 was designed to investigate the correlation between GPA and age of nursing students with the main variables of the study. The results presented in the table using the Pearson correlation coefficient statistical test indicate a negative and significant correlation between GPA and academic procrastination (*r* = -0.424, *p* < 0.001) and a positive and significant correlation between age and perfectionism (*r* = 0.162, *p* = 0.047). Also, age and conversational orientation had a negative correlation (*r* = -0.207, *p* = 0.011).

**Table 2:** Pearson’s correlation between age and GPA with academic procrastination, perfectionism, conversation, and conformity orientation

Variable	GPA (*r*, *p*)	Age (*r*, *p*)
Academic procrastination	*r* = − 0.424, *p* < 0.001^**^	*r* = 0.030, *p* = 0.710
Perfectionism	*r* = − 0.053, *p* = 0.521	*r* = 0.162, *p* = 0.047^*^
Conversation orientation	*r* = 0.104, *p* = 0.204	*r* = − 0.207, *p* = 0.011^*^
Conformity orientation	*r* = − 0.138, *p* = 0.090	*r* = 0.053, *p* = 0.516

^*^p < 0.05; ^**^p < 0.01

The chi-square test (Table 3) shows a significant relationship between gender and the dominant communication patterns in the students’ families (*p* = 0.47). Most female students (79.7%) reported their family’s dominant communication pattern as conversation-oriented. While most male students (64.4%) reported their family’s dominant communication pattern as conformity-oriented.

**Table 3:** Comparison of dominant family communication patterns by gender

Gender	Female		Male		Total		*P*-value
Dominant Family Communication Pattern	Female (n)	female (within Gender%)	Male(n)	Male (within Gender%)	Total (n)	Total (%)	
Conversation orientation	51	79.7	56	64.4	107	70.9	.047^*^
Conformity orientation	13	20.3	31	35.6	44	29.1	
Total	64	100	87	100	151	100	

^*^ Pearson Chi-Square

The results of the study (Table 4) show that there is a positive and statistically significant correlation between perfectionism and academic procrastination among the nursing students studied (*r* = 0.416, *p* < 0.01). Correlation analysis showed a negative and significant relationship between the conversational family communication pattern and academic procrastination (*r* = -0.324, *p* < 0.01). A positive and significant correlation was observed between the conformist family communication pattern and academic procrastination (*r* = 0.262, *p* < 0.01). The findings also showed a negative and significant correlation between perfectionism and the conversational family communication pattern (*r* = -0.336, *p* < 0.01). However, a weak but significant positive relationship was observed between perfectionism and the conformist family communication pattern (*r* = 0.201, *p* < 0.05).

**Table 4:** The correlation matrix between academic procrastination, perfectionism, conversation orientation, and conformity orientation in nursing students

Variable	Academic procrastination	Perfectionism	Conversation orientation	Conformity orientation
Academic procrastination	1	^**^.416	^**^ − .324	^**^.262
Perfectionism	^**^.416	1	^**^ − .336	^*^.201
Conversation orientation	^**^ − .324	^**^ − .336	1	^**^ − .487
Conformity orientation	^**^.262	^*^.201	^**^ − .487	1

^*^p < 0.05

^**^p < 0.01

The results of the study on the relationship between academic procrastination and dominant communication patterns in the families of nursing students, based on the results of the independent T-test, indicate that there is a difference between the average academic procrastination score of students in families with a dominant communication pattern of conversation-centered and families with a dominant communication pattern of conformity-centered (*P* = 0.004). The average procrastination score of students with a conformity-centered family communication pattern is higher than that of students whose dominant family communication pattern is conversation-centered (Table 5).

**Table 5:** Comparison of the average score of academic procrastination with the type of dominant communication pattern in the families of nursing students

	Dominant Family Communication Pattern	Mean	SD	t	df	Sig. (p)
Academic procrastination	conversation orientation	34.65	8.78	-2.95	149	^**^0.004
	Conformity orientation	39.29	8.84			

^**^ Independent t-test

The results of the study on the relationship between perfectionism and dominant communication patterns in the families of nursing students, based on the results of the independent T-test, indicate that there is a difference between the average academic procrastination score of students in families with a dominant communication pattern of conversation-centered and families with a dominant communication pattern of conformity-centered (*P* = 0.000). The average perfectionism score of students with a conformity-centered family communication pattern is higher than that of students whose dominant family communication pattern is conversation-centered (Table 6).

**Table 6:** Mean perfectionism scores by dominant family communication pattern (Independent t-test)

	Dominant Family Communication Pattern	Mean	SD	t	df	Sig. (p)
Perfectionism	conversation orientation	40.50	12.12	-4.05	149	^**^0.000
	Conformity orientation	48.98	10.47			

^**^ Independent t-test

In the model (Table 7), the variables of GPA, perfectionism, family communication patterns, and age were simultaneously entered into the regression equation to determine the contribution of each to predicting academic procrastination. The results presented in the table showed that the model was statistically significant and that all four variables played a significant role in explaining academic procrastination. Based on the standardized coefficients, GPA had the strongest negative predictive power for academic procrastination (B = − 2.399). This result indicates that as GPA increases, the level of academic procrastination significantly decreases. Perfectionism showed a positive and significant relationship with academic procrastination, indicating that higher levels of perfectionism among students are associated with a greater likelihood of procrastination behaviors (B = 0.260). Regarding the conversation orientation, a negative and significant relationship was observed (B = − 0.091). Finally, age was also a negative and significant predictor of academic procrastination (B = − 0.693). The results of the stepwise multiple regression analysis showed that in the first step, GPA alone was able to explain 18% of the variance in academic procrastination (R² = 0.180). With the entry of perfectionism in the second step, the explained variance increased to 33.5% (R² = 0.335), indicating the significant role of perfectionism in improving the predictive model. In the third step, the family communication pattern variable entered the model and increased the explained variance to 36% (R² = 0.36). Finally, with the inclusion of age in the fourth step, the explained variance of the model increased to 38.2% (R² = 0.382), indicating a substantial improvement in the predictive power of the model. In other words, the four variables GPA, perfectionism, family communication pattern, and age together were able to predict approximately 38% of the variance in students’ academic procrastination.

**Table 7:** Stepwise regression results for predicting academic procrastination based on perfectionism and dimensions of family communication patterns

Model	Predictor	B	β	t	*p*
1	Constant	78.297	7.432	---	< .001
	GPA	−2.586	−0.453	−.424	< .001
2	Constant	63.744	71.156	---	< .001
	GPA	−2.459	0.409	−.403	< .001
	Perfectionism	0.290	0.049	−0.395	< .001
3	Constant	69.003	7.371	---	< .001
	GPA	−2.369	0.405	−0.388	< .001
	Perfectionism	0.249	0.052	−0.338	< .001
	Conversation Orientation	−0.079	0.033	−0.170	.017
4	Constant	84.699	10.090	---	< .001
	GPA	−2.399	0.399	−0.393	< .001
	Perfectionism	0.260	0.051	0.354	< .001
	Conversation Orientation	−0.091	0.033	−0.195	.006
	Age	-0/693	0/309	-0/150	0.026

R = 0/618

R Square = 0/382

Adjusted R Square = 0/365

$$\begin{aligned}&Academic\;procrastination\:\\&=\:84.699\;+\;(-2.399\;\times\;GPA)\;\\&+\;(0.260\;\times\;perfectionism)\;\\&+\;(-0.091\;\times\;conversation\;orientation)\;\\&+\;(-0.693\;\times\;age) \end{aligned}$$

## Discussion

The present study aimed to explain and predict academic procrastination based on perfectionism and family communication patterns among nursing students. The findings indicated that nursing students at Jundishapur University of Medical Sciences and its affiliated centers exhibited moderate levels of academic procrastination (Table 1). Studies conducted in Iran, Egypt, and Saudi Arabia are in line with these findings, reporting students’ academic procrastination ranging from moderate to severe. It should be noted, however, that due to differences in measurement instruments, direct comparison of percentages and mean levels of procrastination across studies is difficult [3, 32, 33]. Nursing students, as a potential workforce of the healthcare system, play a vital role in enhancing the quality of healthcare both now and in the future. Their active and responsible presence in clinical settings not only affects patient health but also facilitates the development of their professional skills. Studies indicate that academic procrastination among students is influenced by a range of factors, including individual, educational, and organizational aspects, the nature of the field of study, and the characteristics of academic tasks [8]. Considering academic procrastination as a multifactorial variable, it can be inferred that all of these factors may contribute to its occurrence and persistence. These factors include, but are not limited to, the tendency for immediate gratification during studies, lack of sufficient social support, poor study habits, ambiguous assignments, misalignment of tasks with course objectives, fatigue, ineffective time management, and low self-confidence [8]. Evidence also suggests that family-related factors and personality traits, such as perfectionism and family communication patterns, can act as precursors or moderators of these influences, playing a decisive role in explaining the occurrence of academic procrastination [34, 35]. In this context, the present study indicated moderate to high levels of perfectionism among nursing students at this university. Hill et al., in a large-scale study of university students, found that the prevalence of perfectionism has increased significantly in recent decades, a trend continuously intensified by environmental pressures, academic competition, and social comparisons. The rise of this psychological trait among medical students may be attributed to the competitive, high-pressure, and stressful nature of their educational environments [36]. Moreover, in the current study, perfectionism was positively and significantly associated with academic procrastination (*r* = 0.416, *p* < 0.01). Findings from studies conducted in Pakistan and China are consistent with these results [11, 37]. The primary reason individuals delay tasks they know should be completed, despite potential negative consequences, is often rooted in irrational beliefs such as fear of failure and evaluation anxiety. As noted, maladaptive perfectionists tend to set unrealistically high standards for themselves and believe that outcomes are either perfect or a complete failure [38]. Perfectionism is associated with traits such as fear of criticism, concern over negative judgment, fear of failure, self-doubt, and feelings of guilt and shame, all of which can contribute to increased academic procrastination [34]. The high prevalence of perfectionism among nursing students can be explained by the sensitive, responsibility-driven, and outcome-oriented nature of clinical education in this field. Perfectionism may lead nursing students to engage in excessive self-criticism, thereby increasing stress. In such situations, individuals often choose to avoid confrontations to lessen their anxiety, which leads to increased postponement of actions. However, the relationship between perfectionism and academic procrastination is not always unidirectional or consistent. For instance, a study by Rezaei Gazki et al. in Iran reported no significant association between these two variables [39], reflecting the multifaceted nature of perfectionism and its variable impact on procrastination. It can also be inferred that perfectionists with high personal standards and greater motivation for achievement are more likely to place themselves in challenging situations to reach their goals [40]. Nevertheless, it should be noted that, overall, procrastination primarily arises from failures in self-regulation or self-control [9]. The family is the primary context in which individual behavior is shaped and personality development is guided. Many problems contributing to the occurrence of academic procrastination, such as low self-esteem, lack of self-confidence, and poor resilience, may have their roots in family upbringing and parenting patterns [41]. The present study focused on the dimensions of conversation and conformity. Most participants in this study exhibited a dominant conversation-oriented parenting style (Table 1). The conversation pattern, emphasizing reciprocal interaction, free exchange of ideas, and participation in decision-making, provides a favorable environment for the development of self-efficacy and internal locus of control, factors that are indirectly associated with reduced academic procrastination [29]. This communication style helps children develop greater self-efficacy, internal control, and responsibility, all of which are recognized as negative predictors of academic procrastination. In contrast, in families where interactions are based on conformity, family members are generally encouraged to define their identity within the family framework, with self-concepts largely shaped by the expectations of others. Intergenerational communication in such families is typically grounded in obedience to parents and elders. Families emphasizing conformity often maintain hierarchical structures and are less accepting of children’s independent thinking. This communication style is associated with emotional inhibition, reduced intrinsic motivation, and increased psychological dependence, which can lead to heightened avoidance and procrastinatory behaviors [29]. In the present study, participants with a dominant conversation-oriented pattern exhibited lower average levels of academic procrastination compared to those with a conformity-oriented pattern. Furthermore, dialogue showed a negative correlation with procrastination, whereas conformity showed a positive correlation (Table 4), consistent with the conceptual rationale of these dimensions. However, a previous study reported findings contrary to the present study, with correlations between procrastination and family communication patterns of *r* = 0.682 for conversation and *r* = -0.640 for conformity. This study indicated a direct relationship between academic procrastination and conversation, and an inverse relationship between conformity and procrastination [42]. In interpreting and comparing such findings, it is important to recognize the dynamic and variable influence of psychological factors on individuals. Although conversation-oriented communication is expected to reduce academic procrastination by fostering communication skills, independent decision-making, and responsibility, it may occasionally produce unintended consequences. One possible explanation is the role conflict and academic responsibility challenges arising in contexts of excessive freedom and open decision-making within conversation-oriented families. While children have greater opportunities for expression, experience, and choice, if this freedom is not coupled with training in self-regulation, goal-setting, and internal control, it may lead to task delay and cognitive or behavioral procrastination. Additionally, families with highly conversational children may exercise less direct supervision over academic performance due to high trust in their independence, which can reduce academic structure and increase procrastination [43]. In different cultural contexts, the conversation style may coexist with high performance expectations and parental perfectionism, placing adolescents or students in conflict between their internal desire for self-expression and external pressures to perform flawlessly [42, 43]. Furthermore, conversation-oriented individuals, due to their personality characteristics, exhibited lower average levels of perfectionism compared to those with a dominant conformity pattern in this study (Table 6). This finding suggests that conversation orientation, by providing a conducive environment for the development of adaptive perfectionism, is indirectly associated with reduced academic procrastination, whereas conformity increases the likelihood of maladaptive perfectionism and higher academic procrastination. As previously noted, academic procrastination can result from failures in self-regulation and self-control. In this study, the conformity variable did not play a predictive role in academic procrastination, unlike conversation orientation. This can be interpreted as conversation orientation enhancing self-regulatory skills, which are directly linked to reduced procrastination, while conformity exerts its influence indirectly or interactively rather than directly. Among physiological factors influencing academic procrastination, age also plays a predictive role (Table 7). The present study showed that as individuals’ age increases, the likelihood of academic procrastination also rises. This may be due to increased personal responsibilities, social roles, and developmental pressures, which can affect students’ self-regulatory capacity and increase the propensity for procrastination [44]. Overall, the findings of this study indicate that academic procrastination among nursing students is a multidimensional phenomenon influenced by the interaction of individual, familial, and psychological factors. Perfectionism, particularly in its maladaptive form, and family communication patterns play a decisive role in explaining this behavior. Conversation-oriented parenting can have a protective effect against procrastination by enhancing self-regulation, self-efficacy, and responsibility, whereas conformity, directly or indirectly, and in interaction with traits such as maladaptive perfectionism, fosters procrastinatory behaviors. Given the high-stress and responsibility-driven nature of the nursing profession, identifying these factors can provide a basis for designing effective educational, counseling, and family-based interventions aimed at reducing academic procrastination and improving the academic and professional performance of nursing students. It should also be noted that, although the study limitations are described separately, the results should be interpreted with caution due to the cross-sectional design and the use of self-report instruments.

## Conclusion

Students’ academic procrastination is shaped by both individual traits and family dynamics. Perfectionism contributes to procrastination by fostering pressure and a fear of making mistakes. In contrast, a conversation-oriented family communication style serves as a protective factor, enhancing responsibility and self-efficacy. Conversely, a conformity-based communication style, characterized by rigid expectations and limited autonomy, creates an environment that fosters procrastination. However, the certainty of these findings depends on their relationship with other cognitive, social, and environmental variables.

### Strengths and applications

The strengths of this study include the simultaneous examination of individual and family factors that contribute to academic procrastination. This approach allows for a more comprehensive understanding of the phenomenon by considering the role of perfectionism alongside family communication patterns. By focusing on nursing students, a group with unique educational and clinical challenges, this research enhances the practical value of the findings and their relevance to the medical education system. The results can inform the design of educational and counseling interventions aimed at reducing academic procrastination. Specifically, these interventions can address maladaptive perfectionism and encourage open communication within families. Furthermore, paying attention to these factors can help foster self-regulation, academic responsibility, and professional preparedness among nursing students.

### Limitations

The first limitation of this study pertains to the sample size and statistical population. The research focused on nursing students at Ahvaz Jundishapur University of Medical Sciences. Despite careful sampling, caution should be exercised when generalizing the results to other universities, disciplines, or age groups. Future studies should consider using larger and more diverse samples. While the instruments employed in this study demonstrated good validity and reliability, they primarily relied on self-report methods, which may be influenced by social desirability or perception errors. Incorporating various study methods, such as longitudinal or qualitative approaches, could enhance the validity of the data. Moreover, the regression analyses conducted in this study specifically examined linear relationships and may not capture the complexity of nonlinear relationships or interactions among variables. Additionally, uncontrolled variables such as environmental stress, family conditions, parenting styles, attachment styles, coping strategies, and Internet addiction could account for some of the unexplained variance. Therefore, utilizing methods such as structural equation modeling or path analysis in future research could provide a more accurate and comprehensive understanding of the relationships between variables.

## Data Availability

The datasets generated and/or analysed during the current study are not publicly available due to confidentiality and privacy restrictions, but are available from the corresponding author on reasonable request.
